# Quality improvement program for the severely injured

**DOI:** 10.1007/s00068-025-02826-6

**Published:** 2025-04-29

**Authors:** Isidro Martínez Casas, Ingo Marzi

**Affiliations:** 1 Trauma and Emergency Surgery Unit, General Surgery Department, Virgen del Rocío University Hospital, Sevilla, Spain; 2 Department of Trauma Surgery and Orthopedics, University Hospital, Goethe University Frankfurt, Frankfurt am Main, Germany

**Keywords:** Whitebook, ESTES, Polytruama

## Abstract

Over recent decades, advancements in trauma care have significantly reduced mortality rates among severely injured patients. These improvements are largely attributable to the establishment of trauma care systems, including prehospital management protocols and the creation of trauma centres with immediate surgical team availability. However, patient outcomes continue to vary, reflecting differences in the quality of trauma care influenced by organisational models and local practices. To address this, governments and scientific organisations have underscored the importance of evaluating care quality at local, national, and international levels. This chapter explores strategies for assessing quality of trauma care, establishing reliable quality indicators (QIs), and standardising auditing processes to guide improvements in patient outcomes and system performance.

## Introduction

The World Health Organization (WHO) defines quality of care as “the degree to which healthcare services for individuals and populations increase the likelihood of desired health outcomes and are consistent with current professional knowledge.” This definition underscores two critical concepts: first, that quality of care can and should be measured; and second, that quality is goal-oriented, reliant on clearly defined objectives.

(WHO document) *Guidelines for trauma quality improvement programmes (who.int)*.

Valid and reliable measures of trauma system performance are essential for guiding quality improvement initiatives, benchmarking outcomes, public reporting, investment decisions, and research. Constructing quality indicators (QIs) is a complex process requiring several components, including clear definitions, justification, formulas, data sources, and target populations. Effective QIs should meet four key criteria: importance, usefulness, scientific robustness, and feasibility.

Since the 1970s, when high mortality rates linked to suboptimal trauma care spurred the development of trauma systems and major trauma centres in the United States, preventable deaths and risk-adjusted mortality have become standard metrics for evaluating trauma system and centre performance. However, as preventable mortality rates have declined, these metrics have become less effective as sole indicators of quality. Traditional in-hospital mortality measures also fail to account for the broader continuum of trauma care, including prehospital and post-hospital management, long-term recovery, and the societal and economic impacts of trauma-related disability.

The Donabedian framework offers a conceptual model for evaluating healthcare quality, consisting of three interrelated components:

- Structure: The environment in which care is delivered, including facilities, resources, and organisational characteristics (e.g., trauma centre designations, trauma centre volume, trauma registry availability). While structural indicators are straightforward and objective, their presence does not guarantee quality.
- Process: The methods and activities involved in delivering care (e.g., prehospital times, trauma team activation, adherence to massive transfusion protocols). Process indicators are widely used but require more complex data collection.
- Outcome: The results of care, encompassing metrics such as mortality (e.g., pre-hospital deaths, mortality < 48 h), ICU length of stay, and even patient satisfaction. Outcome indicators are the most challenging to measure but are critical for conditions like trauma, which are characterised by high incidence and severity.

In the 1980s, the American College of Surgeons (ACS) proposed trauma quality indicators for severely injured patients. Subsequent reviews, such as one by Stelfox et al., identified over 1,500 QIs spanning categories including ACS status, patient safety, care outcomes, expert reviews, general auditing, and adherence to guidelines. However, these indicators lack international standardisation and robust evidence supporting their validity and reliability. The development of universally accepted, evidence-based QIs for trauma care remains an urgent priority.

Moreover, trauma care extends beyond the hospital stay, encompassing prevention, post-traumatic management, and societal reintegration. Quality assessment should reflect these broader phases, addressing both direct and indirect costs at national and international levels. Examples include:

- Prevention QIs: Measuring injury risk perception, the impact of public awareness programmes, or psychological consequences in witnesses.
- Post-traumatic Management QIs: Assessing long-term physical and psychological disability support or the tangible costs of care.
- Societal Reintegration QIs: Evaluating career outcomes, psychological outcomes, or outcomes related to support dependency for trauma survivors or witnesses.

A recent international expert panel conducted a web-based consensus survey involving 200 specialists from all WHO regions to evaluate 82 trauma QIs selected from an initial list of 1288. The findings revealed that a globally accepted, evidence-based set of trauma QIs has yet to be established. Current indicators are heterogeneous and inconsistently applied; international collaboration is called for to standardise quality assessment in trauma care.

### Strategies to promote quality and reliability auditing

Severe trauma presents a significant challenge to healthcare systems due to its diversity of presentation, variability in care delivery, frequent deficiencies or errors in care, and the reality that part of the associated mortality remains avoidable. Addressing these challenges requires ongoing quality assessment to identify gaps, implement improvements, reduce morbidity and mortality rates, and enhance survivors’ functional outcomes and quality of life.

Quality improvement relies on continuous education, learning processes, and systematic evaluation of care delivery. Two primary methods for assessing care processes include:

- Medical **Auditing**: A retrospective, systematic analysis conducted by the professionals responsible for providing care.
- **Monitoring**: A continuous and structured quality measurement system that uses predefined quality indicators (QIs) with established optimal benchmarks.

However, a 2009 Cochrane review found no study of sufficient scientific quality to determine whether auditing in trauma care effectively improves outcomes or reduces mortality. While the evidence remains inconclusive, feedback has emerged as a promising tool to improve performance and ensure ongoing quality monitoring.

### The role of feedback in trauma care

Feedback—helpful information or constructive criticism aimed at improving performance—has been shown to enhance surgical and clinical outcomes. Effective feedback mechanisms are integral to continuous monitoring and learning, as demonstrated by several notable examples.

### Case example 1: Ramban model (Haifa, Israel)

A two-part feedback system was implemented to evaluate and improve trauma care:

1. Prehospital Care: Focused on key aspects including airway management, cervical collar application, spinal fixation using backboards, pain assessment and management, and the completeness of prehospital documentation.
2. Hospital Care: Assessed primary hospital-level management including imaging, laboratory investigations, emergency department documentation, electrocardiograms (ECGs), mechanism-of-injury analysis, treatment timelines, and Glasgow Coma Scale (GCS) scoring.

The feedback system, studied over two time periods, demonstrated measurable improvements in trauma patient management. Importantly, feedback was coupled with corrective actions to address cases of mismanagement, ensuring actionable solutions rather than simply identifying issues.

### Case example 2: interhospital feedback in Taiwan

A study by Wang et al. retrospectively analysed data on trauma patients transferred between hospitals across two periods—before and after the introduction of feedback. The feedback approach included:

- Outcome Lectures: Focused on the results of transferred patient care.
- Collaborative Review: Trauma surgeons and emergency department physicians worked together to discuss transfer details, fostering a problem-solving approach rather than assigning blame.

**Fig. 1:**
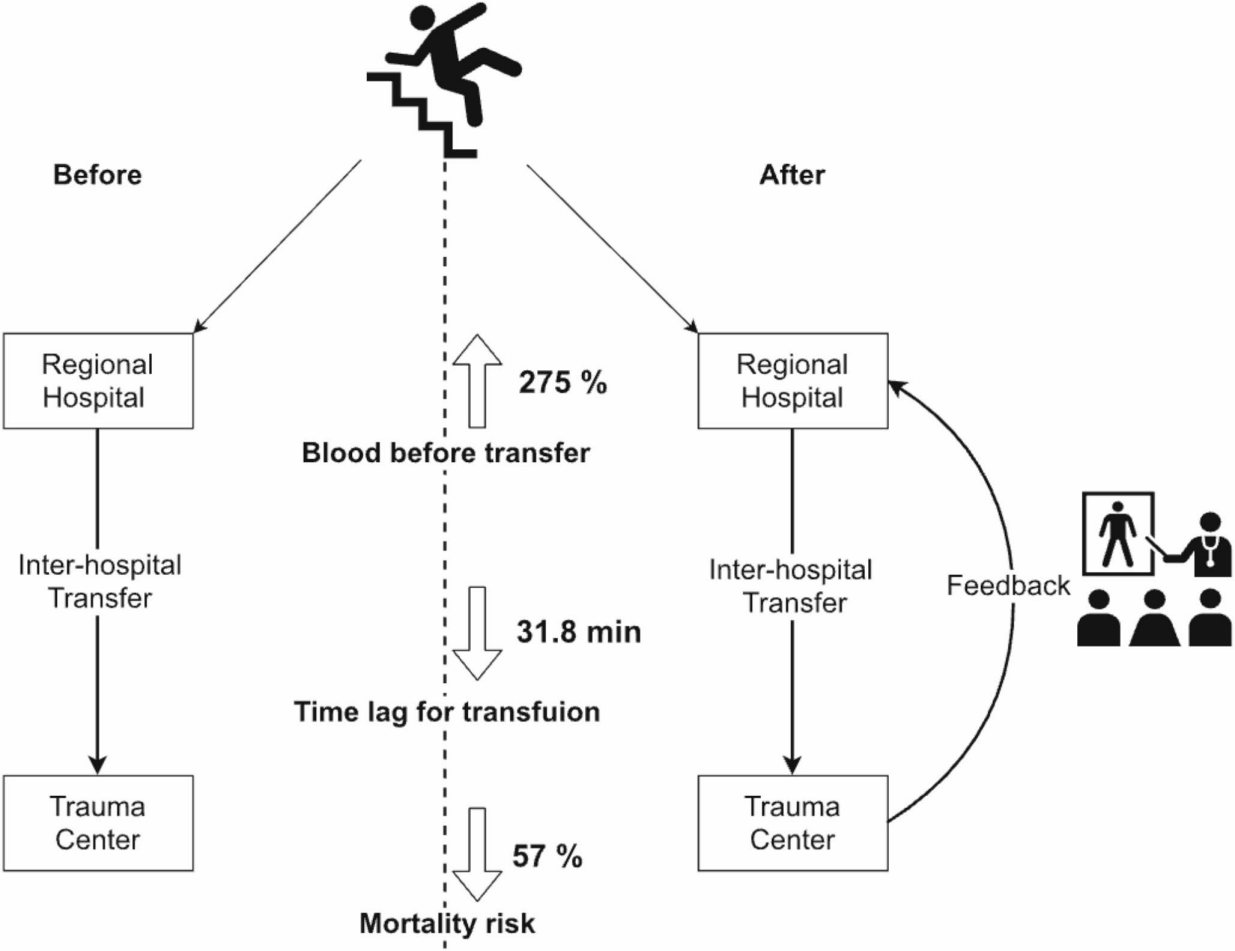
Effect of interhospital feedback on blood transfusion quality in Taiwan. (From Wang CJ et al.)

Breaking the transfer process into smaller steps allowed for targeted problem identification and encouraged cooperation. After adjusting for confounding factors, the feedback-driven period was associated with:

- Higher rates of blood transfusion prior to transfer,
- Shorter time intervals before the first transfusion,
- A marginal reduction in mortality risk (Fig. 1).

### The perfect checklist

The Performance Assessment of Emergency Teams and Communication in Trauma Care (PERFECT) checklist is another notable tool designed to evaluate prehospital trauma training. Developed through qualitative and quantitative analyses and input from experienced academics, clinicians, and emergency medicine trainers, the PERFECT checklist objectively assesses trauma scenarios or real patient care.

The checklist evaluates seven key performance domains essential for clinical competence in trauma care:

1. Primary assessment.
2. Procedures.
3. Technical skills.
4. Trauma communication.
5. Non-technical skills.
6. Global performance.
7. Overall scenario evaluation.

The PERFECT checklist provides a validated and standardised method for assessing prehospital trauma training, ensuring comparability across different scenarios or care settings. In principle, this validated checklist could be used for any prehospital training.

### Trauma registries

Trauma registries (TR) are structured databases containing uniform, consensus-based information collected by experts in trauma care. The primary aim is to provide information that improves the efficiency and quality of trauma care, facilitates epidemiological and clinical research, and supports outcome evaluations. To remain relevant, trauma registries must be adaptable to different healthcare settings and capable of evolving based on findings.

### Data collection challenges

Despite their utility, trauma registries often fail to capture a comprehensive, population-based trauma sample. Many are hospital-based and therefore exclude less severely injured patients or critical cases where death occurs at the scene of the incident. Furthermore, voluntary participation by trauma centres can lead to issues with sample representativeness unless the registry is managed within a single centre framework or a highly organised trauma system.

Accurate quality evaluation hinges on high-quality data, which requires case ascertainment—ensuring all relevant cases are included in the registry. A recent Japanese study comparing the Japan Trauma Data Bank with government evaluation data revealed significant discrepancies, highlighting issues with case ascertainment, coding variability, and data completeness. These inconsistencies undermine the validity of administrative and registry data, which are critical for risk adjustment and outcome evaluation.

In contrast to hospital administrative databases, trauma registries provide more detailed and relevant data for research purposes. However, they often suffer from data quality challenges such as incomplete records, coding inaccuracies, and variability across hospitals.

### Trauma registries in Europe

Europe has developed several trauma registries, ranging from single-centre initiatives to large multicentre networks. Notable examples include:

- *The German National Trauma Registry.*
- *The British Trauma Audit and Research Network (TARN).*
- *The Italian National Registry for Major Injuries.*
- *The Scandinavian Networking Group for Trauma and Emergency Management.*
- The Norwegian National Trauma Registry (NTR).

Efforts to standardise European trauma registries have led to the development of the **Utstein Template for Uniform Reporting of Data Following Major Trauma**. This 35-variable template defines core data required for inclusion and divides variables into three key categories:

- **Predictive Model Variables**: Patient and injury data relevant for outcome prediction.
- **System Characteristic Descriptors**: Data describing system-level differences to enable comparisons.
- **Process Mapping Variables**: Information capturing trauma care processes at individual trauma centres.

While the feasibility of a unified European trauma registry has been demonstrated—particularly through web-based systems requiring minimal additional infrastructure—progress remains limited. Since 2008, collaborative efforts across European trauma professionals have aimed to create a single **European Trauma Registry Network** capable of enabling large-scale, standardised data collection and comparison.

### Ensuring data quality

Validation is critical to ensuring the reliability and utility of trauma registries. Validation can take two forms:

- Internal Validation: Comparison with original data sources to ensure accuracy and consistency.
- External Validation: Ensuring registries capture all relevant cases within the intended population.

Key characteristics for an evaluation of data quality include:

- Accuracy: Exact agreement between datasets.
- Correlation: The association of variables and changes with respect to one another.
- Correctness: Ensuring data falls within acceptable ranges.
- Precision: Specificity of data.
- Consistency: Logical alignment across related data points.
- Completeness: Capturing all required data points and cases.
- Comparability: Standardisation across systems for meaningful comparisons.
- Timeliness: Ensuring data is available when needed.

Few studies have formally evaluated trauma registry quality, and there is currently no universally standardised method for auditing or validating these registries. Establishing clear indicators and reproducible methods for data quality evaluation is therefore essential for improving trauma care.

### Future directions

To enhance the quality of trauma care, international collaboration is essential for developing consensus-based trauma registries and associated quality indicators. These indicators must measure relevant processes, structures, and outcomes, enabling meaningful comparisons and driving improvements in patient care.

As trauma surgeon and author Karim Brohi aptly stated:

Important for this will be legislative policy at national and European levels to support the development of an informatics infrastructure for trauma and the collection of such data on a population-wide basis. However, we must rapidly move towards a whole system approach to data collection, for it is only in the complexity of our trauma patients, and the multitude of interventions they are exposed to, that the true future of trauma care resides.

### Conclusion and needs for the future

The pursuit of high-quality trauma care is a multifaceted endeavour requiring systematic evaluation, continuous feedback, and standardised data collection. Quality indicators play a pivotal role in assessing care processes, benchmarking outcomes, and identifying areas for improvement.

While medical auditing, monitoring, and feedback mechanisms have demonstrated their value, significant challenges remain. The lack of internationally agreed-upon quality indicators and risk adjustment methodologies highlights the need for urgent collaboration among trauma care professionals, policymakers, and researchers.

Trauma registries provide a critical foundation for evaluating care quality and outcomes. However, their effectiveness depends on validation, standardisation, and ongoing refinement to ensure data accuracy and reliability. Moving forward, a unified, consensus-driven approach to trauma data collection at local, national, and international levels must be adopted. This will enable healthcare systems to deliver consistent, evidence-based trauma care and ultimately improve patient outcomes worldwide.

## Data Availability

No datasets were generated or analysed during the current study.
